# Physiotherapists Have Accurate Expectations of Their Patients' Future Health-Related Quality of Life after First Assessment in a Subacute Rehabilitation Setting

**DOI:** 10.1155/2013/340371

**Published:** 2013-11-20

**Authors:** Steven M. McPhail, Emily Nalder, Anne-Marie Hill, Terry P. Haines

**Affiliations:** ^1^Centre for Functioning and Health Research, Buranda Plaza Corner Ipswich Road and Cornwall Street, Buranda, QLD, Australia; ^2^School of Public Health and Institute of Health and Biomedical Innovation, Queensland University of Technology, Kelvin Grove, QLD, Australia; ^3^Rotman Research Institute, Bathurst Street, Baycrest, Toronto, ON, Canada; ^4^School of Physiotherapy, the University of Notre Dame Australia, Mouat Street, Fremantle, WA, Australia; ^5^Southern Health, Allied Health Research Unit, Kingston Centre, Cnr Warrigal and Kingston Roads, Cheltenham, VIC, Australia; ^6^Physiotherapy Department, School of Primary Health Care, Monash University, Monash University Peninsular Campus, VIC, Australia

## Abstract

*Background*. Expectations held by health professionals and their patients are likely to affect treatment choices in subacute inpatient rehabilitation settings for older adults. There is a scarcity of empirical evidence evaluating whether health professionals expectations of the quality of their patients' future health states are accurate. *Methods*. A prospective longitudinal cohort investigation was implemented to examine agreement (kappa coefficients, exact agreement, limits-of-agreement, and intraclass-correlation coefficients) between physiotherapists' (*n* = 23) prediction of patients' discharge health-related quality of life (reported on the EQ-5D-3L) and the actual health-related quality of life self-reported by patients (*n* = 272) at their discharge assessment (using the EQ-5D-3L). The mini-mental state examination was used as an indicator of patients' cognitive ability. *Results*. Overall, 232 (85%) patients had all assessment data completed and were included in analysis. Kappa coefficients (exact agreement) ranged between 0.37–0.57 (58%–83%) across EQ-5D-3L domains in the lower cognition group and 0.53–0.68 (81%–85%) in the better cognition group. *Conclusions*. Physiotherapists in this subacute rehabilitation setting predicted their patients' discharge health-related quality of life with substantial accuracy. Physiotherapists are likely able to provide their patients with sound information regarding potential recovery and health-related quality of life on discharge. The prediction accuracy was higher among patients with better cognition than patients with poorer cognition.

## 1. Background 

Many older adults do not return to their premorbid health states following hospital admissions requiring a period of subacute inpatient rehabilitation [1–4]. Patient-centred models of care are becoming increasingly common among sub-acute rehabilitation facilities [5–7]. Patient-centred models of care may assist older adults recovering from illness or injury to work with their treating team to focus therapy and other treatments on deficits that are most important to the patient to optimise their health-related quality of life at discharge or thereafter [5, 6]. It may therefore be beneficial for patients to have an accurate understanding of their likely recovery in order to make informed decisions about joint therapy goals and when planning for living in the community after discharge [5, 6]. 

There is currently a scarcity of research investigating whether health-professionals' and patients' expectations of future health-states are accurate among patient groups receiving health interventions in settings that utilise patient centred models of care [8]. A recent investigation among older adults in a subacute hospital setting indicated that patients' expectations of their discharge health-related quality of life were moderately accurate [8]. In this context it is likely that older patients will derive information regarding their current health state and potential patterns of recovery from their treating healthcare team [8]. Unfortunately, no previous studies have investigated whether health professionals are able to formulate accurate predictions of older patients' discharge health-related quality of life in subacute hospital settings. 

Physiotherapists are one group of health professionals of particular interest in subacute hospital settings where older adults receive rehabilitative therapies to address functional concerns in order to maximize health-related quality of life on discharge from hospital [2, 9–12]. It is likely that older adults participating in rehabilitative therapies to improve their physical function will derive guidance from their physiotherapists when planning functional goals in this setting. However, there is currently no prior research investigating whether physiotherapists are able to formulate accurate predictions of patients' discharge health states early in a subacute rehabilitation admission. Accurate expectations of their older patients' potential (or lack of potential) to improve their functioning and health-related quality of life during a subacute rehabilitation admission are likely to be beneficial to patients, therapists, and other members of the multidisciplinary team involved in planning clinical interventions as well as planning for discharge [5–8, 11, 13, 14]. 

This study investigated whether physiotherapists' expectations of their patients' discharge health-related quality of life (formulated while conducting their admission assessment) were in agreement with their patients' actual self-reported health-related quality of life on discharge from a subacute rehabilitation unit. Due to the potential influence of patient cognition on self-reported health-related quality of life at discharge, the levels of agreement between physiotherapist predicted health-related quality of life and patients' self-reported discharge health-related quality of life were examined for an entire cohort, as well as two cognition subgroups based on mini-mental state examination scores [15–17]. 

## 2. Methods

### 2.1. Design

A prospective longitudinal cohort design was implemented to examine agreement between physiotherapists' expectations of their patients discharge health-related quality of life versus the patients' actual discharge health-related quality of life as self-reported on the EQ-5D-3L [18]. 

### 2.2. Participants and Setting

A total of 272 patients and their treating physiotherapists (*n* = 23) from a subacute geriatric assessment and rehabilitation unit at tertiary hospital participated in this investigation. Participants were consecutive admissions to this unit following a period of acute management on a specialised hospital ward appropriate for their condition (e.g., an acute orthopaedic ward). The participating unit admits subacute older adults with a range of primary diagnoses for rehabilitative interventions to improve their functioning and prepare them for discharge from hospital. Patients admitted to the participating rehabilitation unit received medical and allied health input designed to maximize functioning, independence, and health-related quality of life on discharge. The typical length of stay in this rehabilitation unit at the time of the study was approximately six weeks. However, length of stay was determined on an individual basis and may have been less or more than six weeks dependent on a range of clinical and external factors.

### 2.3. Outcome Measure

The EQ-5D-3L [18] instrument used to evaluate health-related quality of life has favourable reliability and validity for use with older people [19–24]. This instrument has six questions. The first five items require individuals to rate their health-related quality of life in the domains of mobility, personal care, usual activities, pain and discomfort, and anxiety and depression on a three-level multiple choice scale ((1) no problems, (2) some problems/moderate, and (3) unable/extreme problems). The brief multiple choice scoring system was deemed suitable due to its ease of administration and no requirement for high levels of health literacy in order to understand and respond to each question. The Dolan tariff system [25] was applied to responses to the first five EQ-5D-3L items producing a multiattribute utility score (utility) where death and perfect health are represented by 0 and 1, respectively. Health states considered worse than death are assigned negative values [25]. The sixth and final EQ-5D-3L question is a 100 point Visual Analogue Scale (EQ-VAS) where 0 and 100 are represented by worst and best imaginable health state, respectively [18, 26]. 

The physiotherapists working in the participating subacute rehabilitation unit routinely complete clinical assessments within 72 hours of admission for their patients. It was at the completion of this routine admission assessment that physiotherapists in this unit predicted patients' discharge health-related quality of life. They did this by filling out an EQ-5D-3L selecting the responses they anticipated their patient would self-report during the discharge assessment. 

The Mini-mental state examination (MMSE) was used as a general indicator of patient cognitive ability [16]. The MMSE incorporates a brief assessment of orientation, memory, attention, and arithmetic and is routinely completed for all patients in the participating clinical unit [16, 27]. The MMSE was used to separate patients for subgroup analyses of a poorer cognition group (MMSE of 23 or less) and a better cognition group (MMSE greater than 23) [16, 27]. 

### 2.4. Procedure

On admission and discharge from the unit, patients completed a standard battery of clinical assessments. This assessment included physical performance tests (such as Balance Outcome Measure for Elder Rehabilitation [9]) as well as self-ratings on the EQ-5D-3L to obtain the patient's current perceived health-related quality of life (at admission to the unit). 

Immediately following the completion of the admission clinical assessment battery (but before patients completed their admission EQ-5D-3L self-report), physiotherapy staff completed their discharge prediction rating on the EQ-5D-3L instrument. Physiotherapist participants received a 1-hour training session regarding their role in this study, including how to follow the assessment protocols at the admission and discharge assessments. The training included completing a practice admission and discharge assessment under the supervision of a member of the research team. Additionally, instructions for providing the predicted discharge EQ-5D-3L report were included in each patient's chart. As part of the training session, therapists were instructed to rate the EQ-5D-3L according to how they believed the patient would answer EQ-5D-3L items on discharge from rehabilitation, regardless of whether clinicians agreed with the patient's perspective. 

The MMSE was completed by hospital occupational therapists or medical staff for each patient admitted to the unit. MMSE results along with other patient demographic variables were collated from the medical history, and patients were grouped into a better cognition group (MMSE >23/30) and a poorer cognition group (MMSE ≤23/30) for use in data analysis. 

This research investigation was approved by the local human research ethical review board and complied with the declaration of Helsinki. Patients completed the EQ-5D-3L at discharge as a component of their routine clinical assessments, and gatekeeper consent was provided for physiotherapists' participation in this research.

### 2.5. Analysis

Demographic and clinical characteristics of the sample were tabulated (Table 1). Conventional tests of hypothesis were used to compare demographic characteristics between the lower and higher cognition groups. This included an unpaired *t*-test (age) and a Mann-Whitney *U* test (length of stay in the subacute rehabilitation unit). For the individual EQ-5D-3L domain scores, levels of agreement between physiotherapist predicted discharge rating and actual self-reported discharge EQ-5D-3L responses were calculated using weighted Cohen's Kappa with disagreements of only one level assigned a 0.5 weighting [28] (Table 2); bias corrected 95% confidence intervals for Kappa coefficients were calculated using bootstrap resampling (2000 replications of original sample size, stratifying for cognition grouping where appropriate) [29, 30]. The number (and percentage) of exact matches for each of the domains was also tabulated (Table 2). 

For the summary, EQ-5D-3L scores (utility and EQ-VAS), limits of agreement (LOA) [31], and intraclass-correlation coefficients (Table 3) were calculated separately for patients in each cognition grouping as well as for the total sample. To investigate systematic differences between therapist predicted and patients' discharge health-related quality of life scores (for utility and EQ-VAS) paired *t*-tests were employed separately for each cognition group as well as for the whole patient cohort (Table 3).

## 3. Results 

Overall, 232 (85%) patients had complete data and were included in analyses; 81 (35%) were in the lower cognition group and 151 (65%) were in the higher cognition group. The demographic and clinical characteristics of the sample are presented in Table 1. The poorer cognition group was older by a mean (SD) 7.3 (1.9) years than the better cognition group (*P* < 0.001). The median (IQR) length of stay for the entire sample was 42 (25–66), with no difference between cognition groups (*P* = 0.60). The median (interquartile range) years of experience of physiotherapists (*n* = 23) providing a prediction rating was 6 (2–11). 

The levels of agreement (Kappa statistics and exact matches) between physiotherapist predicted discharge health-related quality of life (EQ-5D-3L domain scores) and patients' self-reported health-related quality of life (EQ-5D-3L domain scores) are reported in Table 2. Kappa coefficients ranged from 0.37 to 0.57 across domains in the lower cognition group and from 0.53 to 0.68 in the better cognition group. Exact agreement ranged from 58% to 83% across domains in the lower cognition group and from 81% to 85% in the better cognition group. Overall, 41% of patients had all five discharge EQ-5D-3L domain responses correctly predicted by their treating physiotherapist at their baseline assessments.

Agreement (intraclass-correlation coefficients and limits of agreement) between physiotherapist predicted discharge health-related quality of life and patients' self-reported health related quality of life at discharge is displayed in Table 3 for the EQ-5D-3L utility and EQ-VAS. The better cognition group and entire sample had narrower limits of agreement and higher intraclass-correlation coefficients than the lower cognition group. No mean differences between predicted and actual discharge EQ-VAS scores were observed for either cognition group or for the entire patient sample as a whole. The mean predicted utility score was lower than the actual discharge utility score for the lower cognition group (−0.09,  *P* = 0.038). No differences were observed in mean utility ratings (predicted versus actual) for the overall sample or for the better cognition group. 

## 4. Discussion 

This investigation revealed substantial agreement between physiotherapists' predictions of their patients' health-related quality of life at discharge and the patients' actual self-reported health-related quality of life at discharge. Interestingly, patient cognitive ability seemed to be a mediating factor in the accuracy of the prediction. The agreement between physiotherapist predicted and self-reported quality of life was not only lower for patients in the poorer cognition group, but physiotherapists tended to underestimate the level of health-related utility (EQ-5D-3L utility) that patients' in the lower cognition group would report at discharge. The size of this underestimation was of a sufficient magnitude to be considered clinically meaningful [32–34]. On the other hand, physiotherapists were able to predict how patients in the better cognition group would report their health-related quality of life at discharge, without any mean overestimation or underestimation and with a substantial degree of accuracy (Table 3). Physiotherapists also had exact matches for all five domain responses for a higher proportion of patients in the better cognition group than the better poorer cognition group (Table 2). 

The purpose of this investigation was not to probe the cause of any underestimation for patients with poorer cognition. However, the authors speculate that there are at least two potential reasons why physiotherapists may have underestimated the summary utility score generated by the EQ-5D-3L instrument for patients with poorer cognitive abilities. First, the physiotherapists would have had some knowledge of patients' cognitive ability from reviewing their medical record and receiving a clinical handover of pertinent information from their colleagues prior to conducting their assessment in this subacute hospital setting. It is plausible that physiotherapists perceived a level of impact from the cognitive impairment on the patients' potential for recovery that was not accurate, and patients subsequently experienced more recovery and better health-related quality of life (EQ-5D-3L utility score) at discharge than the physiotherapists had anticipated. Second, it is also plausible that patients from the poorer cognition group may have lacked some level of insight into their level of functioning when responding to the EQ-5D-3L questions at their discharge assessment. For example, a patient with poor cognition who had some problems with their ability to wash and dress themselves may have reported having no problems with personal care. Potential lack of insight of this nature may have led to difficulty predicting how patients might respond at their discharge assessment rather than difficulty predicting the level of functional recovery a patient may experience relevant to their health-related quality of life [17]. 

There was no clear pattern in the level of prediction accuracy across the five EQ-5D-3L domains for patients in the better cognition group, with the level of exact agreement consistently between 81% and 85% for all five domains. Similarly, there was no clear pattern in the level of prediction accuracy within the poorer cognition group across the five health-related quality of life domains, despite the agreement statistics being lower than those observed for the better cognition group. This was an interesting finding, given that some prior research has indicated that proxies may be better able to predict how patients will respond to health survey questions for more observable domains like mobility than for less easily observable domains like depression [35–37]. One could argue that physiotherapists, who have particular expertise in physical recovery, may have been more likely to predict health-related quality of life domains related to physical functioning than depression or anxiety. However, such an assertion would not have been supported by the empirical data from this investigation. 

There are several important implications for patient centred models of care in subacute rehabilitation settings from this research. Patients that experience debilitating injuries or illnesses are likely to derive information about their potential for recovery from their treating health professionals when planning goals and making joint treatment decisions intended to maximize their health-related quality of life on discharge and thereafter [5, 6]. Physiotherapists are likely to be a reliable source of information regarding patients' potential for recovery during the subacute rehabilitation period from as early as immediately following their initial assessment on admission to subacute rehabilitation units. However, further research is warranted to investigate whether clinicians tend to underestimate the potential for recovery among patients with poorer cognition or the extent to which patients with poor cognition may under-report potential deficits in their health-related quality of life when completing questionnaires.

There may be other explanatory factors beyond the scope of this investigation that influenced patient self-reports among some patients and contributed to some discrepancies between predicted and actual health-related quality of life reports in both cognition groups. Some previous reports have demonstrated paradoxical improvements in self-reported health-related quality of life as people have aged despite declining physical functioning and increased depression [38–40]. Changes in the way people reconceptualize, reprioritize, and recalibrate their health state (ratings) as an effect of natural adaptation over time have been termed “response shift” and may have influenced the older adults in this population receiving rehabilitation [7, 24, 41]. Individual factors, such as these, that may cause a measurement related bias potentially influencing patient reports of health-related quality of life (and difficulty anticipating future health state reports) are worthy of consideration as a future research direction with relevance to patient centred models of care. 

There are several strengths and limitations for this research. First, all participating physiotherapists and patients were from a single geographical location. Physiotherapists from other geographical locations may not have responded in the same way. On the other hand, the inclusion of 23 different physiotherapists and 224 patients provides some ability to generalize findings to other similar populations. Second, only the EQ-5D-3L instrument was used. While this instrument is among the most widely used generic health-related quality of life measures, is suitable for use among older adults [19, 23], and is suited to this kind of predictive study due to the clearly delineated response options, use of another method of health-related quality of life reporting may not have yielded the same levels of predictive accuracy. Another important caveat is that this investigation only included patients in the subacute inpatient rehabilitation phase of their recovery. It is possible that physiotherapists answered some of the domain responses knowing the minimum level of functioning that would be required for patients to be able to be safely discharged and provided predictive responses congruent with minimum requirements for safe discharge to community living rather than basing their specific responses on knowledge of the pathology, the patient, and their prognosis. Nonetheless, this investigation was successful in addressing the research aims. 

It may be useful for future research in this field to examine the prediction of health state outcomes across transition periods. This may include transitions from inpatient rehabilitation to returning to live in the community, particularly among older patients at risk of requiring institutional care. Intervention decisions in the subacute setting may influence the likelihood of subsequent hospital or nursing care admissions. The ability for clinical teams to be able to inform their patients about the likelihood of functional abilities and health-related quality of life in the post-discharge period may assist patients (in conjunction with their treating teams) to make sound decisions regarding postdischarge care arrangements. Additionally, specific physiotherapist-related factors that may have influenced their ability to predict patients' discharge health-related quality of life were beyond the scope of this investigation. However, future investigations of health-professional attributes that may influence their ability to anticipate their patients' future health states may also be worthy of consideration to advance this field of research. 

## 5. Conclusion

Physiotherapists in this subacute rehabilitation setting predicted their patients' discharge health-related quality of life with substantial accuracy. They are likely to be able to provide their patients with sound information regarding the potential recovery and health-related quality of life on discharge. The prediction accuracy was higher among patients with better cognition than patients with poorer cognition. Physiotherapists tended to underestimate the self-reported discharge health-related quality of life of patients in the poorer cognition group by a small, but potentially clinically meaningful, margin. 

## Figures and Tables

**Table 1:** Participant demographic and clinical information.

	Low cognition group (*n* = 81)	High cognition group (*n* = 151)
Mean (SD) Age	79.0 (11.8)	71.7 (14.9)
Gender—Female (%)	51 (63%)	88 (58%)
Clinical diagnosis category at admission (%)		
Orthopedic	30 (37%)	45 (30%)
Stroke	12 (15%)	41 (27%)
Other Neurological	11 (14%)	24 (16%)
Geriatric deconditioning	12 (15%)	14 (9%)
Other disabling condition requiring rehabilitation	16 (20%)	27 (18%)
Median (IQR) days length of stay	45 (25–65)	42 (26–70)
EQ-5D-3L at admission		
Mean (SD) Utility Score	0.444 (0.402)	0.425 (0.352)
Mean (SD) VAS	63 (19)	57 (19)

**Table 2:** Agreement (Kappa coefficients and exact match) between physiotherapist predicted and patient self-reported discharge EQ-5D-3L domain responses.

	Agreement per domain Kappa (95% CI)					Exact match number (%)					
	Mobility	Personal care	Usual activities	Pain/discomfort	Anxiety/depression	Mobility	Personal care	Usual activities	Pain/discomfort	Anxiety/depression	All domains correct
Lower cognition (*n* = 81)	0.51 (0.37, 0.67)	0.42 (0.25, 0.59)	0.37 (0.23, 0.53)	0.54 (0.36, 0.72)	0.57 (0.34, 0.76)	59 (73%)	53 (65%)	47 (58%)	62 (77%)	67 (83%)	24 (30%)
Better cognition (*n* = 151)	0.68 (0.57, 0.79)	0.67 (0.55, 0.79)	0.64 (0.51, 0.75)	0.67 (0.56, 0.79)	0.53 (0.36, 0.69)	126 (83%)	129 (85%)	123 (81%)	127 (84%)	128 (85%)	72 (48%)
Combined (*n* = 232)	0.62 (0.52, 0.71)	0.57 (0.47, 0.67)	0.52 (0.42, 0.62)	0.63 (0.53, 0.72)	0.55 (0.41, 0.67)	185 (80%)	182 (78%)	170 (73%)	189 (81%)	195 (84%)	96 (41%)

**Table 3:** Intraclass-correlation coefficient (ICC), mean EQ-5D-3L utility and Visual Analogue Scale (VAS), and limits of agreement (LOA) between physiotherapist predicted and actual discharge health-related quality of life reports (*n* = 232).

	Measure	ICC (95% CI)	Anticipated mean (95% CI)	Actual mean (95% CI)	Limits of agreement			*P* value*
					Lower LOA (95% CI)	Mean difference (95% CI)	Upper LOA (95% CI)	
Lower cognition	EQ-5D-3L utility	0.50 (0.31, 0.65)	0.669 (0.608, 0.729)	0.757 (0.698, 0. 815)	−0.611 (−0.553, −0.669)	−0.088 (−0.030, −0.146)	0.434 (0.493, 0.376)	0.038∗
	EQ-5D-3L VAS	0.49 (0.31, 0.64)	75.1 (72.3, 78.0)	77.5 (74.3, 80.8)	−25.3 (−28.3, −22.2)	2.40 (−0.677, 5.47)	30.0 (27.0, 33.1)	0.271
Better cognition	EQ-5D-3L utility	0.75 (0.67, 0.81)	0.765 (0.734, 0.795)	0.748 (0.714, 0.782)	−0.267 (−0.291, −0.244)	0.017 (−0.006, 0.040)	0.301 (0.278, 0.324)	0.469
	EQ-5D-3L VAS	0.75 (0.68, 0.82)	79.1 (77.4, 80.7)	79.0 (77.0, 81.1)	−16.1 (−17.4, −14.8)	−0.40 (−1.34, 1.26)	16.0 (14.7, 17.3)	0.976
Combined	EQ-5D-3L utility	0.62 (0.53, 0.69)	0.731 (0.702, 0.760)	0.751 (0.721, 0 .781)	−0.417 (−0.443, −0.391)	−0.020 (−0.046, 0.006)	0.377 (0.351, 0.403)	0.351
	EQ-5D-3L VAS	0.64 (0.56, 0.71)	77.7 (76.2, 79.2)	78.5 (76.8, 80.2)	−20.1 (−21.5, −18.8)	0.810 (−0.564, 2.19)	21.8 (20.4, 23.1)	0.482

*P value < 0.05 indicated that a systematic difference exists (i.e., predicted discharge health-related quality of life was consistently higher or lower than the actual self-report at discharge).
